# Intravenously administered interleukin-7 to reverse lymphopenia in patients with septic shock: a double-blind, randomized, placebo-controlled trial

**DOI:** 10.1186/s13613-023-01109-w

**Published:** 2023-03-12

**Authors:** Thomas Daix, Armelle Mathonnet, Scott Brakenridge, Pierre-François Dequin, Jean-Paul Mira, Frederique Berbille, Michel Morre, Robin Jeannet, Teresa Blood, Jacqueline Unsinger, Jane Blood, Andrew Walton, Lyle L. Moldawer, Richard Hotchkiss, Bruno François

**Affiliations:** 1 Réanimation Polyvalente, INSERM CIC 1435 and UMR 1092, CHU Limoges, Limoges, France; 2 Médecine Intensive Réanimation, CH Orléans, Orléans, France; 3 Department of Surgery, Sepsis and Critical Illness Research Center, University of Florida College of Medicine, Gainesville, FL USA; 4 Médecine Intensive Réanimation, INSERM U1100 Centre d’Étude des Pathologies Respiratoires and INSERM CIC 1415, CHRU Tours and Université de Tours, Tours, France; 5 Réanimation Médicale, Assistance Publique des Hôpitaux de Paris, Groupe Hospitalier Universitaire de Paris Centre, Hôpital Cochin, and Faculté de Médecine, Université Paris Descartes, Paris, France; 6 RevImmune, Bethesda, MD USA; 7 INSERM CIC 1435 and UMR CNRS 7276, INSERM 1262, CHU Limoges and Faculté de Médecine, Université de Limoges, Limoges, France; 8 Department of Anesthesiology, Washington University School of Medicine, St Louis, MO USA; 9 Present Address: Department of Surgery, Harborview Medical Center, University of Washington, Seattle, WA USA

**Keywords:** Sepsis, Immune restoration, Lymphocyte, Interleukin-7

## Abstract

**Background:**

Profound lymphopenia is an independent predictor of adverse clinical outcomes in sepsis. Interleukin-7 (IL-7) is essential for lymphocyte proliferation and survival. A previous phase II study showed that CYT107, a glycosylated recombinant human IL-7, administered intramuscularly reversed sepsis-induced lymphopenia and improved lymphocyte function. Thepresent study evaluated intravenous administration of CYT107. This prospective, double-blinded, placebo-controlled trial was designed to enroll 40 sepsis patients, randomized 3:1 to CYT107 (10 µg/kg) or placebo, for up to 90 days.

**Results:**

Twenty-one patients were enrolled (fifteen CYT107 group, six placebo group) at eight French and two US sites. The study was halted early because three of fifteen patients receiving intravenous CYT107 developed fever and respiratory distress approximately 5–8 h after drug administration. Intravenous administration of CYT107 resulted in a two–threefold increase in absolute lymphocyte counts (including in both CD4^+^ and CD8^+^ T cells (all *p* < 0.05)) compared to placebo. This increase was similar to that seen with intramuscular administration of CYT107, was maintained throughout follow-up, reversed severe lymphopenia and was associated with increase in organ support free days (OSFD). However, intravenous CYT107 produced an approximately 100-fold increase in CYT107 blood concentration compared with intramuscular CYT107. No cytokine storm and no formation of antibodies to CYT107 were observed.

**Conclusion:**

Intravenous CYT107 reversed sepsis-induced lymphopenia. However, compared to intramuscular CYT107 administration, it was associated with transient respiratory distress without long-term sequelae. Because of equivalent positive laboratory and clinical responses, more favorable pharmacokinetics, and better patient tolerability, intramuscular administration of CYT107 is preferable.

*Trial registration*: Clinicaltrials.gov, NCT03821038. Registered 29 January 2019, https://clinicaltrials.gov/ct2/show/NCT03821038?term=NCT03821038&draw=2&rank=1.

**Graphical Abstract:**

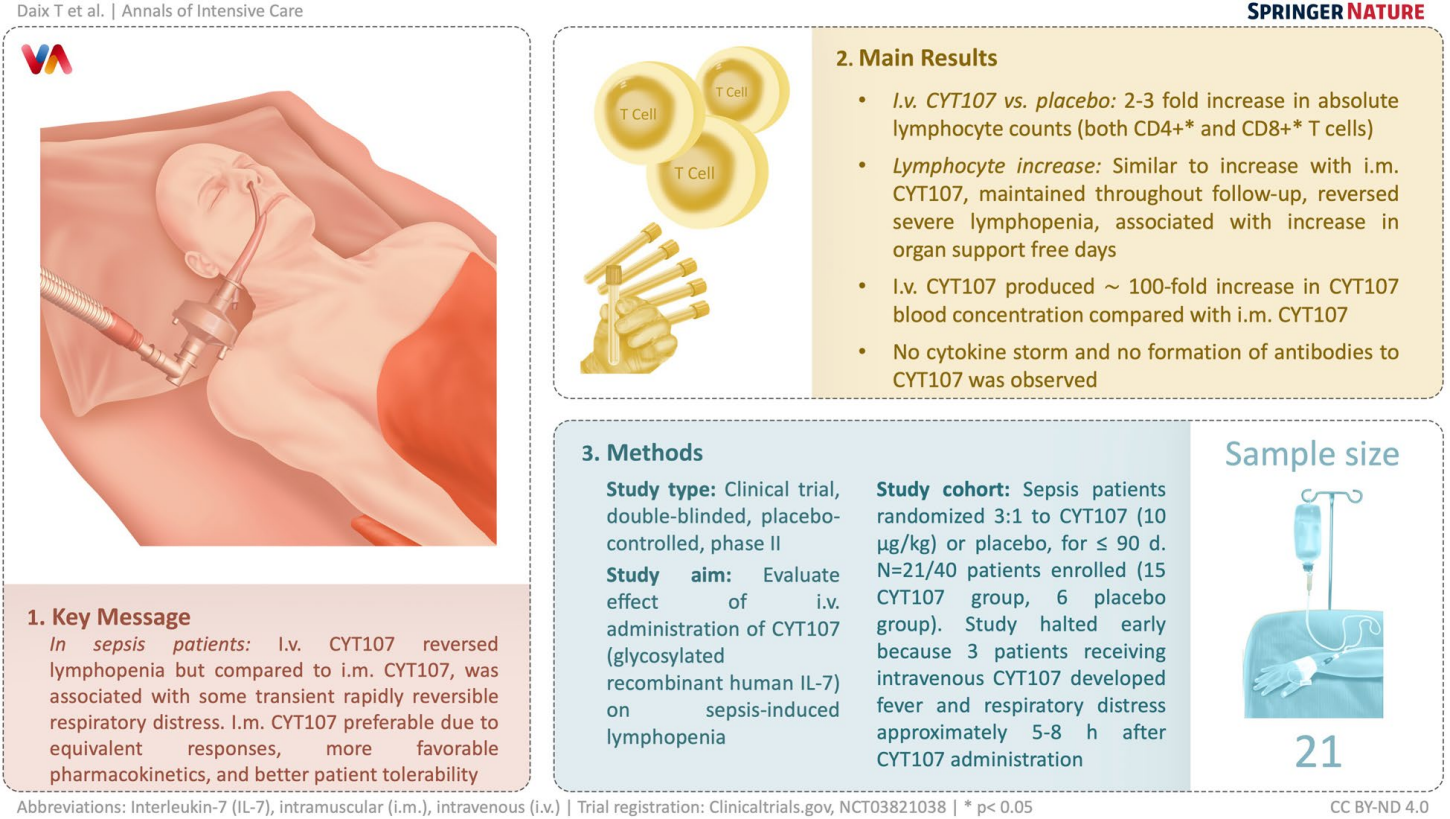

**Supplementary Information:**

The online version contains supplementary material available at 10.1186/s13613-023-01109-w.

## Background

A pathophysiologic hallmark and likely a key mechanism of immune suppression in sepsis is a profound apoptosis-induced depletion of lymphocytes [1–5]. Postmortem examinations of patients who died of sepsis show extensive lymphocyte depletion occurring in spleens, intestines, and lungs [1, 4]. The tissue lymphocyte depletion occurring during sepsis is paralleled by a decrease in the absolute number of circulating lymphocytes, i.e., lymphopenia [5–11]. This sepsis-induced lymphopenia is broad-based and includes CD4^+^ and CD8^+^ T cells, B cells, and natural killer cells [1, 9]. Lymphocytes are required for an effective host defense against invading microbes. Studies in bone marrow or solid organ transplantation patients demonstrate a close correlation between low CD4^+^ T cell counts and development of new opportunistic infections and mortality [12]. Similarly, studies show that septic patients with more severe and persistent lymphopenia are more likely to die versus septic patients who are either not lymphopenic or who have a lesser degree of lymphopenia [5–8]. The contention that sepsis-induced lymphopenia plays a causative role in sepsis-induced morbidity and mortality is supported by numerous independent investigators who documented that prevention of sepsis-induced lymphopenia by a variety of independent methods improved survival in clinically-relevant animal models of sepsis [3, 13]. Interleukin-7 (IL-7) is a lymphocyte growth factor that is essential for lymphocyte survival and proliferation. Acting via the JAK/STAT pathway, IL-7 induces lymphocyte proliferation and prevents lymphocyte apoptosis by increasing the anti-apoptotic molecule Bcl-2 and decreasing the pro-apoptotic molecule Bim [8, 9]. A previous study by our group, conducted in a small cohort of patients with sepsis, showed that CYT107, a glycosylated recombinant human IL-7, administered intramuscularly was well-tolerated, and reversed the sepsis-induced lymphopenia by causing a two–threefold increase in the absolute lymphocyte counts (ALC) compared to patients who received placebo [14]. CYT107 not only increased the number of circulating lymphocytes but also increased lymphocyte activation consistent with an ability of CYT107 to ameliorate the T cell dysfunction that characterizes sepsis-induced immunosuppression [15, 16]. A common consequence of the intramuscular administration of CYT107 was a grade 2–3 injection site reaction consisting of local erythema and warmness [14].

The purpose of this study was to determine the pharmacokinetics and pharmacodynamics of intravenous administration of CYT107 in patients with sepsis. An additional outcome goal was to examine whether intravenous administration of CYT107 would increase ALC and organ support-free days, and would obviate the potential injection site reactions that occur with intramuscular administration. In this manner, we sought to gain further insight into the safety and efficacy of IL-7 as a potential immune adjuvant in the therapy of sepsis-induced lymphopenia. Here, we report the results of a prospective, multicenter, randomized, double blind, placebo-controlled phase IIb trial of intravenously administered CYT107 in patients with vasopressor dependent sepsis and severe lymphopenia.

## Methods

### Study design

This was a prospective, multicenter, randomized, double blind, placebo-controlled phase IIb trial of CYT107 in patients with vasopressor-dependent sepsis and severe persistent lymphopenia (two ALCs ≤ 900 lymphocytes/µL). Patients received intravenously CYT107, 10 µg/kg of ideal body weight or placebo (randomized 3:1) twice a week over 3 weeks. CYT107 was administered through a central or peripheral intravenous catheter over one to two minutes at a non-diluted concentration of 2 mg/mL. The control group received the same volume of normal saline solution.

The study was undertaken at eight intensive care units in France and two in the United States and registered on clinicaltrials.gov (NCT03821038). The study was conducted in accordance with the ethical principles of the Declaration of Helsinki and the International Council for Harmonization Guidance for Good Clinical Practice, and was approved by the relevant local French and American ethics committees. Patients or their legal representatives gave written informed consent prior to inclusion in the trial.

### Patient selection—septic shock, organ dysfunction, and lymphopenia

Patient entry criteria were as previously described [14]. Briefly, patients 18–85 years of age who met Sepsis-2 criteria [17], who had vasopressor-dependent septic shock (defined as hypotension requiring vasopressor treatment for at least 6 h to maintain a systolic blood pressure ≥ 90 mmHg or a mean blood pressure ≥ 65 mmHg), and either acute respiratory failure requiring mechanical ventilation and/or acute kidney injury (creatinine > 2.0 mg/dL or urine output < 0.5 mL/kg/h fr > 4 h despite adequate fluid resuscitation) were eligible for study inclusion. In addition, prior to study enrollment, patients had to have persistent lymphopenia defined as two ALCs ≤ 900 cells/mm^3^ at least 12 h apart and within 48 h after the diagnosis of sepsis.

#### Exclusion criteria

Patients were excluded for evidence of autoimmune disorders, active hematological diseases, cancer with current chemo or radiation therapy, treatment with corticosteroids equivalent to a dose ≥ 300 mg/day of hydrocortisone, and treatment with immunosuppressive medications.

### Data collection

The primary outcome was change in ALC at day 29 between the placebo and treatment groups. Secondary outcomes were: (1) number of safety events within 90 days, (2) incidence of secondary infections within 90 days, (3) number of organ support free days alive (OSFDa) during index hospitalization, (4) effect of CYT107 on CD4^+^ and CD8^+^ T cells, monocyte HLA-DR expression, and cytokine concentrations and (5) CYT107 pharmacokinetics analysis. Of note, the a priori design of this phase IIb study was not statically powered for any of the clinical outcomes.

Plasma CYT107 concentrations and pharmacokinetic assessments were evaluated during the first (day 1) and after the fifth injection (day 15) at 1, 3, 5, 7 and 9 h post injection. Cytokine concentrations (TNF-α, IL-6 and IL-10) were measured at the same time points as CYT107 via an ELISA test as previously described [14] (see Additional file 1). CD3^+^, CD4^+^ and CD8^+^ T cells as well as monocyte HLAD-DR expression were analyzed weekly until day 29 and again at day 90 using Duraclone™ technology (Beckman Coulter, Indianapolis, IN, USA). Data were analyzed with either FlowJo™ Software v10.2 (FlowJo™ Software, Becton, Dickinson and Company, Franklin Lakes, NJ, USA) or Kaluza Software v2.1 (Beckman Coulter, Indianapolis, IN, USA). Flow cytometry methods are detailed in Additional file 1.

Adverse events (AEs) and serious adverse events (SAEs) were reported until day 90. Grade 3 AEs were declared as SAEs. An unblinded Data Safety Monitoring Board (DSMB) including five independent experts reviewed the data after the first 12 patients and at additional time points if requested by the Steering Committee.

All secondary infections that occurred within 90 days were identified by the investigators and blindly adjudicated secondarily by three independent experts as previously described [14]. During the ICU stay, organ support data, i.e., for mechanical ventilation, vasopressor support and/or renal replacement therapy, and antibiotic consumption were also collected. Antibiotic consumption was defined as the number of days of antibiotic therapy, regardless of the antibiotic, from day 0 to Day 29 or hospital discharge.

### Statistical analysis

Enrollment of 40 ICU patients was planned to be randomized in a 3:1 ratio to include at least 30 evaluable subjects receiving CYT107. Descriptive statistics for baseline characteristics and study outcomes are presented in aggregates and stratified by treatment arm, and separately shown for the modified-intention-to-treat and safety analysis. Continuous variables are presented as median and interquartile range (IQR). Categorical variables are summarized using sample proportions and the associated 95% confidence intervals.

A simple inferential analysis was implemented for certain study outcomes. Categorical outcomes were evaluated using Fisher’s exact test. Continuous outcomes and especially absolute lymphocyte count were analyzed with linear mixed models for repeated measures incorporating treatment group, measurement day, and hour within day, when appropriate. Other continuous variables were compared using Wilcoxon non parametrical test. A natural-log transformation was applied to continuous variables to adjust for skewness when necessary. Unadjusted *t*-tests were used to test pairwise comparisons of interest.

## Results

### Study population and outcome

Twenty-one patients were enrolled (fifteen CYT107, six placebo) at eight French and two US sites. The Steering Committee decided to stop enrollment early, prior to the planned goal of 40 patients, on recommendations of the DSMB due to an unexpected pharmacokinetic profile of CYT107 and safety concerns. The twenty-one patients who were enrolled had a median age of 69 (57, 76) years and median SOFA score of 9 (5, 11) (Table 1). The main sites of infection were pulmonary (n = 8) and intra-abdominal (n = 5). The severity scores were similar in both groups. At baseline, patients had low and comparable ALCs (0.6 (0.4, 1.0) vs 0.6 (0.3, 0.9)10^3^ cells/µL for CYT107-treated and placebo-treated patients, respectively; *p* = 0.57). Baseline CD 4^+^ and CD8^+^ T cell numbers were also similar for CYT107-treated and placebo-treated patients, (0.326 (0.185, 0.673) vs 0.637 (0.164, 0.703); *p* = 0.52 and 0.112 (0.050, 0.216) vs 0.134 (0.028, 0.170); *p* = 0.97, respectively) (Table 1). HLA-DR expression on monocytes (m-HLA-DR) was low in both groups, and not statistically different.

**Table 1:** Patient characteristics

	Treatment group (n = 15)	Placebo group (n = 6)	p
Age^a^	70 (46, 76)	63 (57, 77)	0.97
Sex-ratio	6.5	2	0.54
Baseline APACHE II^a^	19 (14, 20)	15 (9, 19)	0.22
Baseline SOFA Score^a^	8 (5, 10)	10 (7, 11)	0.56
Source of infection, (n (%)) - Pulmonary - Intra-abdominal - Blood stream - Skin and soft tissue - Endocarditis - Urinary tract	5 (24%) 5 (24%) 2 (10%) 2 (10%) 1 (5%) 0	3 (14%) 0 2 (10%) 0 0 1 (5%)	
Creatinine, (mmol/L)^a^	115 (78, 224)	155 (80, 553)	0.54
White blood cell, (G/L)^a^	15 (10, 20)	15 (12, 15)	0.47
Lymphocytes, (G/L) ^a^	0.6 (0.4, 1.0)	0.6 (0.3, 0.9)	0.57
CD4 T cells, (10^3^cells/µL)^a^	0.326 (0.185, 0.673)	0.637 (0.164, 0.703)	0.52
CD8 T cells, (10^3^cells/µL)^a^	0.112 (0.50, 0.216)	0.134 (0.28, 0.170)	0.97
mHLA-DR, (mAB/cell)^a^	4153 (2490, 8010)	7679 (3938, 13,481)	0.38
Neutrophils, (G/L)^a^	12 (9, 18)	12 (10, 13)	0.57
Monocytes, (G/L)^a^	0.5 (0.4, 1.0)	0.8 (0.7, 0.8)	0.82
Platelets, (G/L)^a^	130 (58, 256)	87 (74, 96)	0.40

^a^Values reported are median (interquartile range)

Five patients (24%) died before day 90, two out of six patients in the placebo group (33%) and three out of 15 patients receiving CYT107 therapy (20%). The adjudication committee identified secondary infections in two of the six patients (33%) in the placebo group and in eight of the fifteen (53%) of the CYT107 treated patients;* p* = 0.64. ICU length of stay was longer in the CYT107 group vs placebo, i.e., (16 (9, 32) vs 14 (9, 19) days respectively, but this was not statistically significant, *p* = 0.54). Patients receiving CYT107 had more organ support free days alive compared to patients receiving placebo (19 (12, 29) vs 8 (3, 11) days; *p* < 0.05) (Table 2).

**Table 2:** Population outcomes

	Treatment group (n = 15)	Placebo group (n = 6)	p
During ICU stay: - Vasopressor support, (day)^a^ - Mechanical ventilation, (day)^a^ - Renal replacement therapy, (n)	0 (0, 2) 8 (5, 12) 6	0 (0, 7) 7 (0, 14) 3	0.83 0.54 1
Organ support free days (day)^a^	19 (12, 29)	8 (3, 11)	< 0.05
Secondary infection, (n(%))	8 (53.3%)	2 (33.3%)	0.64
Site of secondary infection (n = 10 (%)): - Pulmonary - Bloodstream - Other^b^	4 (40.0%) 1 (10.0%) 3 (30.0%)	1 (10.0%) 1 (10.0%) 0	
Length of ICU stay, including ICU readmission (day)*	16 (9, 32)	14 (9, 19)	0.54
Antibiotic consumption at day 29 or hospital discharge, (day)^a^	20 (4, 29)	13 (11, 16)	< 0.05
Mortality at day 90, (n(%))	3 (20.0%)	2 (33.3%)	0.48

^a^Values reported are median (interquartile range). Dead patients are counted as 0 organ support free days alive

^b^ Skin, intra-abdominal, osteitis

### Primary outcome

**Fig. 1:**
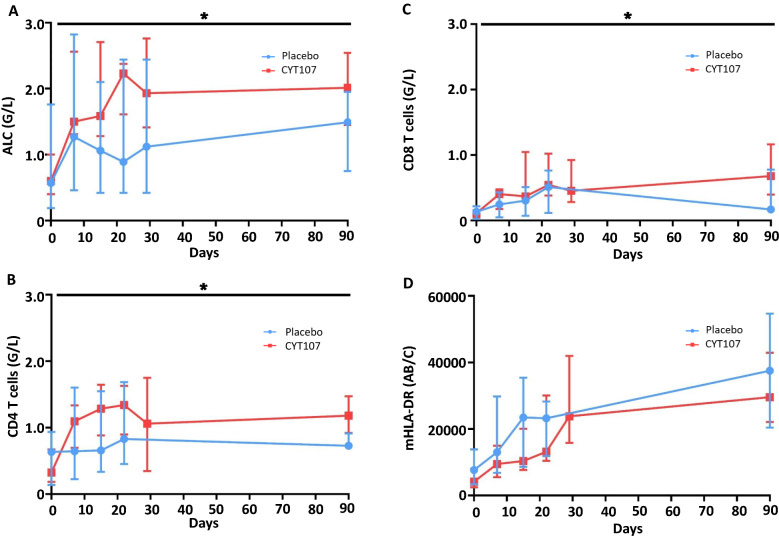
CYT107 increases absolute lymphocyte counts and both CD4^+^ and CD8^+^ T lymphocytes*.* Patients’ peripheral blood was analyzed at day 0, 7, 15, 22, 29 (or hospital discharge) and day 90. Data are represented in median and interquartile. Patients who received CYT107 are represented in red. Patients who received placebo are represented in blue. **A** *Median absolute lymphocyte counts*: The absolute lymphocyte counts were higher in patients treated with CYT107 versus placebo; *p = 0.005.** B** and **C** represents the median CD4^+^ and CD8^+^ T cells count for each group, respectively, as obtained by flow cytometry. Both CD4^+^ and CD8^+^ T cell counts were significantly higher in the CYT107- versus placebo-treated group after day seven, *(*p* < 0.005).** D** HLA-DR expression on CD14^+^ monocytes was measured by flow cytometry and was indicated in median number of antibodies bound per cell (Ab/cell)

Patients receiving intravenous CYT107 had a larger increase in their ALC compared to patients receiving placebo (Fig. 1A). Indeed, the treatment group effect was significant (*p* = 0.005; F(1,19) = 10.40), as well as the time effect *(p* < 0.001; F(10,159) = 6.14) and the interaction (*p* = 0.003; F(10,159) = 2.83) indicating different trajectories for the rise in ALC in CYT107- versus placebo-treated patients. More specifically, both CD4^+^ and CD8^+^ T-cell counts were significantly higher in the CYT107- versus placebo-treated group after day seven (*p* < 0.005). CD8^+^ T cell counts remained significantly increased out to day 90, while CD4^+^ T cell counts at day 90 closely approached statistical significance, (*p* = 0.06), (Fig. 1B, C and Additional file 1: Figures S1, S2). IL-7 did not affect monocyte HLA-DR expression (Fig. 1D).

### Safety and tolerability of intravenous CYT107

Twelve of the fifteen septic patients who received study drug and six of six of the placebo-treated septic patients had no observable adverse effects of CYT107 or placebo respectively on their clinical signs or symptoms. Specifically, there were no detectable changes in body temperature, heart rate, respiratory rate, pulse oximetry, or respiratory parameters following CYT107 or placebo administration. Three patients treated with CYT107 developed fever and respiratory distress occurring approximately 5–8 h after drug administration. It was the investigators’ opinions that the events were possibly drug-related because of the temporal association of occurring following CYT107 administration. Two of the three patients were treated with inhaled bronchodilators and non-invasive ventilation with prompt resolution of their symptoms. One of the three patients required intubation and mechanical ventilation for respiratory distress. This patient’s respiratory distress rapidly resolved and he was extubated approximately 12 h later. All three patients returned to baseline respiratory status, recovered from their sepsis, and were discharged from the hospital. None of these 3 patients received any additional doses of CYT107 after initial reaction.

**Fig. 2:**
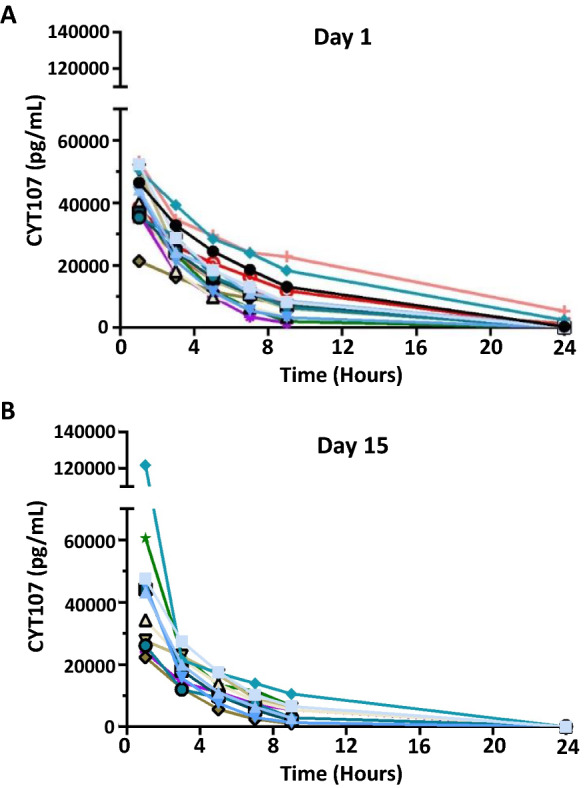
CYT107 Pharmacokinetics. Concentration of CYT107 was measured in patients’ plasma at 1, 3, 5, 7, 9 and 24 h after intravenous administration. Values reported are the pharmacokinetics results for each patient who received CYT 107 at day 1 in** A** n = 15 and at day 15** B** n = 12

After enrollment of the first 12 patients, the first recommendation of the DSMB was to continue enrollment in the IRIS 7CD trial. Following occurrence of respiratory events, the opinion of the DSMB was requested again and it was decided to immediately perform the pharmacokinetic analysis of the available samples. On day 1 (Fig. 2a), pharmacokinetic analysis of study drug revealed an unexpectedly high circulating concentration of CYT107 (mean C_max_ at 41,423 pg/mL) one hour after injection The CYT107 concentration subsequently decreased steadily over the next 24 h. On day 1, the area under the curve (AUC) was 273,772 pg/mL/h. On day 15, the pharmacokinetics analysis of CYT107 was quite similar, with a mean C_max_ at 41,454 pg/mL and AUC at 194,026 pg/mL/h following injection (Fig. 2b). Because of unexpectedly high circulating concentration of CYT107 following intravenous CYT107 administration and the possible adverse effect of CYT107 to induce transient respiratory distress, the Steering Committee, upon the recommendations of the DSMB, decided to proceed to an early stop of enrollment at 21 patients (15 in the treatment group and 6 in the placebo group) and to unblind the study.

Immunogenicity analyses for the detection of antibodies against the recombinant human CYT107 were performed at day 0 and 29 and on days 90 and 180 if they had been positive at day 29. However, all patients treated with CYT107 were negative for antibody detection at day 29.

### Impact of CYT 107 on cytokines

**Fig. 3:**
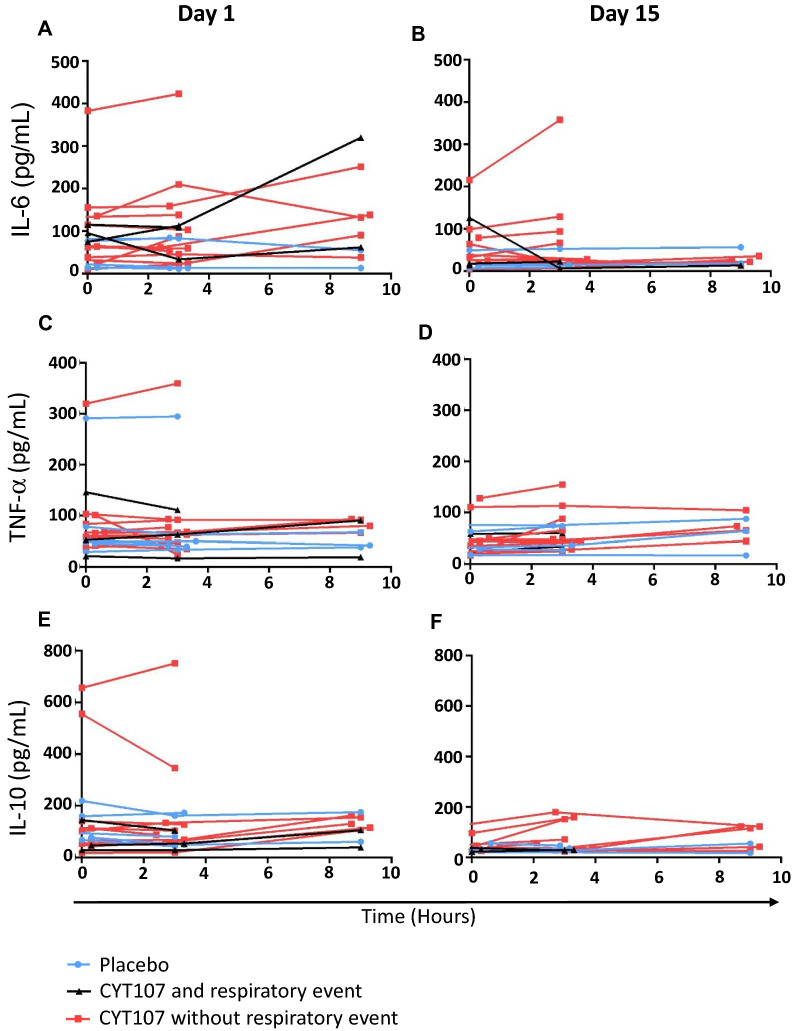
Effect of CYT107 on circulating cytokines. Plasma concentration at day 1 and day 15 of the pro-inflammatory cytokines IL-6 (**A**, **B**) and TNF-a (**C**, **D**) and the anti-inflammatory cytokine IL-10 (**E**, **F**). Placebo group individuals are in blue. Patients that received CYT107 and had a respiratory event are in black. Patients that received CYT107 and did not have a respiratory event are in red

Analysis of circulating cytokines IL-6, IL-10, TNF-α at day 1 and day 15 demonstrates that CYT107 does not lead to major increases in their concentration in blood (Fig. 3). Also, the three patients who developed respiratory distress showed no large increase in the levels of either of these three cytokines (Fig. 3) compared to other CYT107-treated patients or placebo-treated patients.

## Discussion

The present study confirms and extends findings from our earlier investigation [14] demonstrating that administration of a glycosylated recombinant human IL-7 (CYT107) can effectively reverse the profound sepsis-induced lymphopenia that is a defining pathophysiologic hallmark of sepsis [1–5, 15, 16]. CYT107 acts to enhance host immunity by restoring both the number and function of CD4^+^ and CD8^+^ T cells that are massively depleted in patients with sepsis and that play a critical role in host defenses against microbial pathogens [14]. The potential importance of CYT107’s effect to restore lymphocyte effectors in septic patients, is underscored by numerous studies showing a direct correlation between lymphopenia and increased incidence of sepsis-induced morbidity and mortality [5–8]. Furthermore, a growing number of case reports in which CYT107 was used on a compassionate basis in lymphopenic patients with life threatening viral and fungal infections showed that CYT107 reversed the lymphopenia, decreased viral and or fungal load, and was associated with recovery [18–21]. The effect of IL-7 to reverse sepsis-induced lymphopenia occurs via two independent mechanisms. IL-7 blocks sepsis-induced lymphocyte apoptotic cell death by increasing the anti-apoptotic molecule Bcl-2 [8, 13]. Secondly, IL-7 increases lymphocyte proliferation via its effects to activate the PI3 kinase pathway [8].

Additional hallmarks of immune suppression in sepsis are impaired T lymphocyte interferon gamma (IFN-γ) production and decreased monocyte HLA-DR expression. IL-7 restores T cell IFN-γ production as documented in both animal models of sepsis, in ex vivo blood studies from septic patients, and in blood samples from septic patients who were treated with IL-7 on a compassionate basis [8]. Decreased monocyte HLA-DR expression has been correlated with increase morbidity and mortality in sepsis [8]. The decrease in monocyte HLA-DR expression in sepsis may impair monocyte antigen presentation capability and thereby decrease host response to the invading pathogens. Previously, we reported a non-statistically significant trend (*p* = 0.07) for IL-7 to increase monocyte HLA-DR expression in patients with sepsis who were treated with CYT-107 versus placebo [14].

Although both intravenous and intramuscular administration of CYT107 reversed sepsis-induced lymphopenia, the pharmacokinetic data show that intravenous administration of CYT107 produces an approximate 50-to-100-fold increase in the circulating concentration of the drug compared to subcutaneous or intramuscular administration. This marked difference in circulating concentrations of CYT107 with intravenous versus intramuscular administration is consistent with other drugs that undergo target-mediated clearance [22–24]. IL-7 is presumed to be removed from the circulation by IL-7 receptor expressing lymphocytes which have a high affinity but low capacity mechanism of clearance [25, 26]. After intravenous administration of CYT107, the target mediated clearance mechanisms are presumably saturated, and the drug accumulates in the blood. Thus, pharmacokinetic modeling showed us that lowering the dose would not resolve this issue, while after intramuscular administration the receptor mediated clearance remains accessible to the smoother profile of IL-7 release in the blood. Interestingly, target-mediated clearance has also been reported for IL-15 which, like IL-7, is a member of the common gamma chain cytokine family. Patients had a 10–30 fold higher circulating concentration of an IL-15 super agonist when the drug was administered by the intravenous route compared to the subcutaneous route [27]. Despite the higher circulating concentration of the IL-15 super agonist occurring with intravenous IL-15 drug delivery, the effect of the IL-15 super-agonist to increase natural killer cells was comparable by both drug delivery methods, an effect similarly observed in the present study of CYT107. Additional reports of target-mediated drug disposition have been reported for growth factors, and antibodies [22–24].

Despite this major increase in circulating levels of CYT107 in patients treated by intravenous versus intramuscular route, the pharmacodynamic effect of intravenous CYT107 to increase patients’ absolute, CD4^+^ and CD8^+^ lymphocyte counts was similar to the effect observed in septic patients who were treated with intramuscular CYT107 [14]. Both intravenous and intramuscular CYT107 increased the ALC over baseline by approximately two–threefold. It is important to note that the effect of CYT107 to increase the patients’ ALCs persisted for weeks after completion of drug administration as has been previously reported in patients treated with CYT107 [14].

Intravenous administration of CYT107 was associated with a small number of respiratory distress events that ultimately led to the early termination of the study. Importantly, these events were transient and resolved without any apparent long-term sequelae. Interestingly, respiratory distress secondary to CYT107 administration has not been observed in over 500 patients who have been treated with subcutaneous or intramuscular CYT107 therapy to date [28–33]. For example, in a previous trial of 27 patients in septic shock, intramuscular administration of CYT107 was well tolerated and there was no development of or worsening of existing fever, tachycardia, hypotension, or respiratory difficulty in any of the seventeen patients who received CYT107 [14]. Similarly, twelve critically-ill mechanically ventilated patients with COVID-19 and severe lymphopenia who were treated on a compassionate basis with intramuscular injections of CYT107 exhibited no changes in temperature, heart rate, respiratory rate, oxygen saturation, ventilatory requirements, or other clinical signs or symptoms [34].

We suspect that the 50–100 fold higher plasma concentration of CYT107 that occurs following intravenous administration compared to intramuscular or subcutaneous administration is responsible for the transient respiratory difficulty that occurred in three of the fifteen patients in the present study. The fact that there was no large increase in circulating IL-6 or TNF-α in the three patients who developed respiratory difficulty after CYT107 administration suggests that a systemic cytokine storm was not a likely mechanism for their respiratory distress. We speculate that the transient respiratory difficulties were likely due to trafficking of lymphocytes to the lung and subsequent recruitment of other immune effector cells with resulting transient local inflammation within the lung. IL-7 causes upregulation of multiple adhesion molecules on lymphocytes that help traffic lymphocytes from the circulation to sites of inflammation or infection [35, 36]. During infection, endothelial cells upregulate the corresponding receptors for lymphocyte-associated adhesion molecules and assist in trafficking of lymphocytes to help combat invading microbes.

The effect of IL-7 to induce lymphocytes to leave the bloodstream and migrate to sites of infection is most readily observed by the rapid fall in the ALC that occurs within hours after IL-7 administration [14, 35, 36]. This early transient *decrease* in the ALCs with IL-7 precedes the subsequent sustained *increase* in the ALC that subsequently occurs. In this regard, our group previously reported an approximate 30% *decrease* in the blood ALCs at 24 h following subcutaneous or intramuscular CYT107 administration in patients with sepsis [14]. We also previously reported that CYT107 therapy led to dramatic recruitment of CD3^+^ lymphocytes to the site of intractable fungal infection which was associated with rapid resolution of the life-threatening infection that had been resistant to all other therapies [20]. We speculate that this early effect of IL-7 to direct lymphocytes to areas of infection is inherently beneficial by helping to contain and eliminate the microbial pathogens, but may be excessive when IL-7 plasma concentrations are dramatically (100-fold) elevated when administered intravenously.

## Conclusion

Similar to the effect of intramuscular CYT107, intravenous administration of CYT107 reversed the profound sepsis-induced loss in CD4^+^ and CD8^+^ T cells in sepsis. The effect of CYT107 to restore and subsequently maintain lymphocyte counts continued for several weeks after cessation of drug therapy. However, intravenous CYT107 administration resulted in plasma concentrations approximately 50-to-100 times higher than that reported after intramuscular administration, and some transient respiratory distress. Therefore, intravenous administration of CYT107 should be avoided and intramuscular administration should be the preferred route in future clinical studies.

## Supplementary Information

**Additional file 1: Figure S1.** Flow cytometry strategy for T cells analysis and absolute count. **Figure S2.** CYT107 versus placebo effect displayed separately for each individual patient.

**Additional file 2.** Collaborators list.

## Data Availability

The datasets used and/or analyzed during the current study are available from the corresponding author on reasonable request.

## Abbreviations

AE: Adverse event
ALC: Absolute lymphocyte count
DSMB: Data safety monitoring board
ICU: Intensive care unit
IL: Interleukin
SAE: Serious adverse event
