# Supplementary material for: Intravenously administered interleukin-7 to reverse lymphopenia in patients with septic shock: a double-blind, randomized, placebo-controlled trial
Source: Ann Intensive Care. 2023 Mar 12;13:17. doi: 10.1186/s13613-023-01109-w (PMC10008152; doi:10.1186/s13613-023-01109-w)
Supplement: Supplementary file 1 — Additional file 1: Figure S1. Flow cytometry strategy for T cells analysis and absolute count. Figure S2. CYT107 versus placebo effect displayed separately for each individual patient. [file 13613_2023_1109_MOESM1_ESM.docx]

**Intravenously Administered Interleukin-7 Reverses Lymphopenia in Patients with Septic Shock - a double-blind, randomized, placebo-controlled trial**

**Supplementary data**

**Cytokine analysis:** Circulating cytokines IL-6, IL-10 and TNF-α were analyses from plasma via ELISA. Cytokine kits for TNF-α, IL6, and IL-10 were obtained from Biolegend and R&D Systems assays were performed as per the manufacturer’s instructions. Analysis were done on MAGPIX System (DiaSorin Company, Saluggia, VC, Italia

**Flow Cytometry analysis:** 100µl of total peripheral blood were pipetted in a test tube containing a cocktail of dried antibody (Custom design Duraclone tube with CD3-FITC, HLA-DR-PE, CD127-PC7, CD38-APC, CD4-PB and CD8-KrO, all from Beckman Coulter, Indianapolis, IN, USA) and a fixed known number of beads. Sample were incubated at room temperature for 15min in the dark. Then 900µl of red blood cells lysis buffer (BD FACS lysing solution, Becton, Dickinson and Company, Franklin Lakes, NJ, USA) were added in the tube for another 15 min of incubation and samples were ready for acquisition on a flow cytometer. T cells were gated on SSC and CD3 then the absolute number of CD3+, CD3+CD4+ and CD3+CD8+ events were calculated according to the following formula: (number of events of interested record/number of beads record)*(total number of beads/100).

For HLA-DR analysis, fresh peripheral blood sample (<90min after drawing) were stain with BD Quantibrite Anti-HLA-DR/Anti-Monocyte cocktail (BD Biosciences, Becton, Dickinson and Company, Franklin Lakes, NJ, USA) following previously described protocol (Hamada et al cyto b 2021). Briefly, 25µl of whole blood were stain with 10µl of Quantibrite HLA-DR/Monocyte cocktail for 30 min at room temperature in the dark. Then, sample were lysed for 10 min at room temperature in the dark with BD FACCS lysis buffer (BD Biosciences, Becton, Dickinson and Company, Franklin Lakes, NJ, USA) and wash with PBS. Cells were resuspended in PBS and analyzed on a flow cytometer. Absolute number of HLA-DR receptor on the cells surface was calculated with the help of BD Quantibrite beads (BD Biosciences, Becton, Dickinson and Company, Franklin Lakes, NJ, USA) according to manufacturer instruction.

**Supplemental Figure 1**. *Flow cytometry strategy for T cells analysis and absolute count.*

Cell count (cells/µL) = (Number of cell events/Number of beads events)x(Total number of beads per test/Volume of specimen in µL)

CD4+ T cells count=(3473/4400)x(109746/100)=866 cells/µL or 0.866 cells/GL

The first panel indicates how CD3 T cells and Beads were selected according to their fluorescence in FITC. A discriminant was use to excluded from the acquisition all FITC negative event. Beads aggregates were excluded using an histograms and the number of beads singulet was use for calculation of absolute number. From the CD3+ T cells gating, on the bottom row, doublet were excluded. Then classical representation of CD4 and CD8 T cells was used to obtain the number of event acquired for each population. Then the indicated formula was used to retrieve the absolute number of CD4 and CD8 T cells. An example of the calculation for CD4+ T cells is given. Cell count (cells/µL) = (Number of cell events/Number of beads events)x(Total number of beads per test/Volume of speciment in µL)

CD4+ T cells count=(3473/4400)x(109746/100)=866 cells/µL or 0.866 cells/GL

**Supplemental Figure 2.** *CYT107 versus placebo effect displayed separately for each individual patient.*


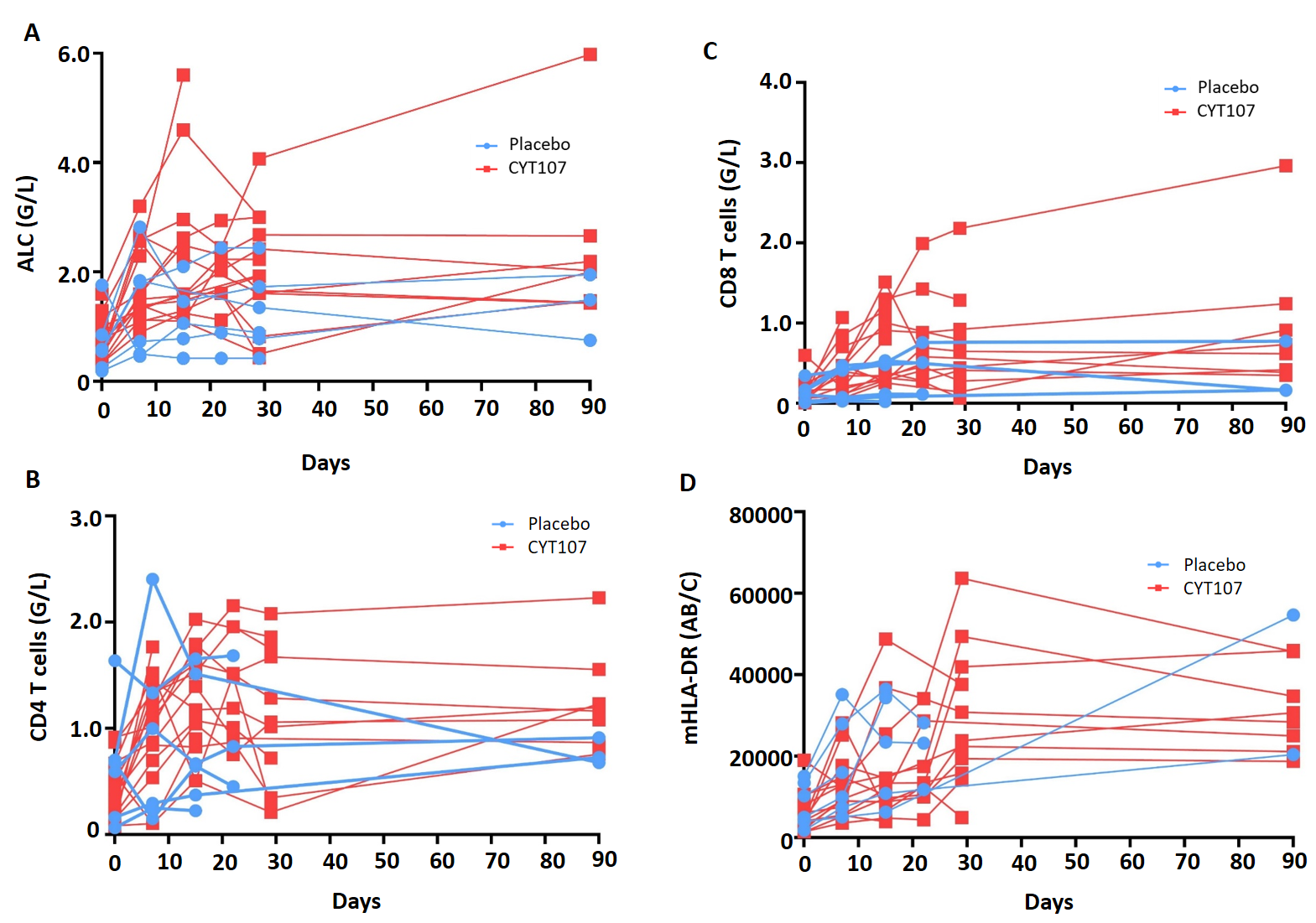


A) Change in absolute lymphocyte counts; B) change in CD4 T cells; C) change in CD8 T cells; D) change in monocyte HLA DR expression; AB/C is number of antibodies bound per cell x10^4^ (monocyte).
