# Supplementary material for: Intravenously administered interleukin-7 to reverse lymphopenia in patients with septic shock: a double-blind, randomized, placebo-controlled trial
Source: Ann Intensive Care. 2023 Mar 12;13:17. doi: 10.1186/s13613-023-01109-w (PMC10008152; doi:10.1186/s13613-023-01109-w)
Supplement: Supplementary file 2 — Additional file 2. Collaborators list. [file 13613_2023_1109_MOESM2_ESM.pdf]

Intravenously Administered Interleukin-7 to Reverse Lymphopenia in Patients with Septic Shock - a double-blind, randomized, placebo-controlled trial

Collaborators list

CHU de Limoges, France :

VIGNON Philippe; EVRARD Bruno; GOUDELIN Marine ; DESACHY Arnaud; VAIDIE Julien ;

CHU d'Angers, France :

ASFAR Pierre; MERCAT Alain; BELONCLE François; PIERROT Marc; SOUDAY Vincent; KOUATCHET Achille; MORTAZA Satar; MEZDAD Tin-Hinan; DEMISELLE Julien; MAHIEU Rafaël; JULIEN Hélène; LEMERLE Marie; OLIVIER Pierre Yves

Hôpital Cochin, AP-HP, Paris, France :

CARIOU Alain; CHARPENTIER Julien; CHICHE Jean-Daniel; PENE Frederic; JAUBERT Paul; DUPLAND Pierre; JOZWIAK Mathieu; AUBOURG Frédérique; NGUYEN Lee; AIT HAMOU Zakaria; GAVAUD Auriane; BENGHANEM Sarah;

Hôpital Henri Mondor, AP-HP, Créteil, France :

DE PROST Nicolas ; MEKONTSO DESSAP Armand; CARTEAUX Guillaume; RAZAZI Keyvan; BAGATE François; PERIER François; BENDIB Ines; BENELLI Brice;

HAUDEBOURG Anne Fleur; BENAIS Morgan; MASI Paul; ARRESTIER Romain; TUFFET Samuel; BERINGUER Hélène;

CHU de Dijon, France :

QUENOT Jean-Pierre ; ANDREU Pascal ;. JACQUIER Marine ; LABRUYÈRE. Marie

Hôpital Edouard Herriot, HCL, Lyon, France:

RIMMELE Thomas ; TURC Jean; CARABALONA Jean-Francois;

CHR d'Orléans, France :

BOULAIN Thierry ; BARBIER François; BRETAGNOL Anne; MULLER Grégoire; NAY Mai Anh; RUNGE Isabelle; SKARZYNSKI Marie; JACQUIER Sophie; BENZEKRI-LEFEVRE Dalila;

CHU de Tours, France :

EHRMANN Stéphan; MERCIER Emmanuelle; GAROT Denis; LEGRAS Annick; JOUAN Youenn; SALMON GANDONNIERE Charlotte; MORISSEAU Marlène; GUILLON Antoine; BODET COTENTIN Laetitia; PEREZ Yonatan; MANKIKIAN Stéfan; BESSE Marie Catherine;
